# Patent citation network analysis: A perspective from descriptive statistics and ERGMs

**DOI:** 10.1371/journal.pone.0241797

**Published:** 2020-12-03

**Authors:** Manajit Chakraborty, Maksym Byshkin, Fabio Crestani

**Affiliations:** Faculty of Informatics, Universitá della Svizzera italiana, Lugano, Switzerland; The Bucharest University of Economic Studies, ROMANIA

## Abstract

Patent Citation Analysis has been gaining considerable traction over the past few decades. In this paper, we collect extensive information on patents and citations and provide a perspective of citation network analysis of patents from a statistical viewpoint. We identify and analyze the most cited patents, the most innovative and the highly cited companies along with the structural properties of the network by providing in-depth descriptive analysis. Furthermore, we employ Exponential Random Graph Models (ERGMs) to analyze the citation networks. ERGMs enables understanding the social perspectives of a patent citation network which has not been studied earlier. We demonstrate that social properties such as homophily (the inclination to cite patents from the same country or in the same language) and transitivity (the inclination to cite references’ references) together with the technicalities of the patents (*e.g.,* language, categories), has a significant effect on citations. We also provide an in-depth analysis of citations for sectors in patents and how it is affected by the size of the same. Overall, our paper delves into European patents with the aim of providing new insights and serves as an account for fitting ERGMs on large networks and analyzing them. ERGMs help us model network mechanisms directly, instead of acting as a proxy for unspecified dependence and relationships among the observations.

## Introduction

A patent is a contract between the inventor or assignee and the state, granting a limited period of time to the inventor to exploit his invention. The reasons for patenting could be myriad, ranging from the elementary need for exclusive rights to a particular technology or invention to building a positive image of an enterprise. In the context where they apply, patents are vital for technological innovation. Patents often incentivize synergistic partnerships between companies and academic institutions. They are known to be used for generating revenues for patent assignees and are often used as tools for competitive market advantages. Patents are also the basis for productive activities within and outside of firms engaged in services and other important business sectors. An invention is a solution to a specific technological problem and is either a product or a process. So, essentially patents are important not only for the protection of intellectual rights but also in solving a wide category of technological problems and promoting innovation. Usually, patents are associated with economic growth, but in certain cases, such as in a time of economic crisis, they can prove to be detrimental to such growth. While there have been a lot of studies and research carried out on academic research papers [1], the patents have not been the subject of such a rigorous study on the same scale despite the fact that the history of patents dates back to the thirteenth century [2].

Patent citations are references to already existing technology within either patents or scientific literature based on which the current patent is modeled. They bear a resemblance to references in academic research papers. These references are primarily concerned with older patents (patent-to-patent citations) on which the current one is build to prove novelty or for continuity (“prior art”) and, generally to a lesser extent, to non-patent items (non-patent references, NPRs), particularly academic and scientific publications (scientific non-patent references, SNPRs). The onus of including relevant references in academic and scientific publications is on the authors. However, in the case of patents, both inventors, as well as patent examiners, are equally responsible [3]. Having said that, there are significant differences between patent citations and scientific ones. As pointed out by Meyer [4], there are both organisational influences, legal and strategic factors and differences in patent examination offices that dictates which patents are cited. Often a patent not only contains a solution to a problem but also highlights opportunities for applications. In addition, journal articles do not emphasise the deficiency in earlier undertakings as frequently or as rigorously as in patents. Accounting for all these factors in understanding which patents are cited and why is beyond the scope of our study. Hence, we do acknowledge that while there are some parallels between patent and scientific citations, there are several unique differences as well. In this paper, our objective is solely focused on trying to understand which factors can possibly affect formation of citations from a network structure point of view, without accounting for extraneous factors.

In the patent application process, patent examiners suggest missing citations to applicants to ensure full coverage of related works so as to avoid patent infringement issues. There are basically two kinds of citations: *forward* citation and *backward* citation. Forward citations concern patents which cite a particular patent while backward citations are patents that are cited by a specific patent. Often, citation analysis is performed over citation graphs to identify similar works or to measure the impact factor of journals, researchers *etc*. With respect to academic citations, numerous methods have been proposed for computing these scores, such as bibliographic coupling [5], co-citation [6] or the Hirsch h-index metric [7]. References to prior patents *i.e.,* patent citations and the state-of-the-art included therein, along with the frequency with which prior documents are cited are regularly used as indicators for estimating the commercial and technological value of a patent. Depending on the nature of the technology, patent citations are often used to identify “key” or pivotal patents.

Global trends for transferring technology could also be inferred from patent data. The geographical regions that a patent is granted in, demonstrates the wide applicability of the technology as perceived by the inventor. Based on the fact that protection for an invention may be sought in multiple countries, the Organisation for Economic Co-operation and Development (OECD) developed a proxy measure of technology transfer [8]. This stems from the notion that inventors and organizations would not be interested in filing patent applications in more than one country unless there is a market potential for the technology proposed in the patent in those particular countries.

There are several factors that determine how innovation evolves in a particular geographical location over a period of time, which includes political, social, environmental, and judicial policies, among others. While it is nearly impossible to chart all the factors and measure their impact on innovation, investigating how innovation grows and affects the knowledge flow across countries and classes, irrespective of such influences is still very important. Previous studies, like the one by Acs *et al.* [9], suggested that patents provide a fairly reliable measure of innovative activity. According to Trajtenberg [10], apart from serving as indicators, patent citations represent the causal relationships between citing and cited patents reinstating the view that innovation is a continuous and incremental process. All of these necessitates a closer look at patent citation networks, especially with respect to how citations are formed and their relevance in imparting knowledge about factors influencing them. The literature on patent citations is vast, and numerous studies have been conducted on different aspects, *e.g.,* ethnicity [11, 12], social networks [13], geographic proximity [14, 15] and so on. Instead, in this paper we aim to study the patent citation network from the perspective of citation forming mechanisms and the factors that influence them. This is a novel perspective on the problem.

In this paper, provide an extensive study of the patent citation network from a statistical viewpoint. In particular, we carry out experiments to understand citation formation mechanisms in patents. We extract comprehensive information on patent meta-data such as assignee, language, country among others, and employ it in Exponential Random Graph Models (ERGMs) [16], which are well established statistical models for the analysis of network data. For our study, we carry out the analysis with the European (EPO) patents from MAREC dataset (http://www.ifs.tuwien.ac.at/imp/marec.shtmlhttp://www.ifs.tuwien.ac.at/imp/marec.shtml). The patents in this collection have been aggregated and curated within the period of 1976-2008 in several languages. Thus the contributions in the paper are as follows:

- We provide a methodology based on descriptive analysis of the patent citation network to gain a shallow understanding of the network structure and its implications.
- We carry out an in-depth study of the citation network among top patent applicants to verify whether the “small-world” effects holds true in this context. This also acts as a case-study to identify hubs and authorities within the sample network, thus enabling us a deeper understanding of how top companies interact among themselves in terms of patent citations.
- We employ ERGMs on the patent citation network to study the effect of various self-defined covariates on the patent citation forming mechanisms. We posit that since the patent network is a large network consisting of several nodes and edges, ERGMs will be able to estimate parameters effectively. To the best of our knowledge, such a study focusing on patent citation forming mechanisms using ERGMs does not exist in the literature that deals with the effects of factors like the influence of patent recency, overlapping categorization and so on.

The paper is organized as follows: In the section titled *Related Works*, we discuss past citation network studies with special emphasis on patents and delineate our contributions to the literature. The section titled *Exponential Random Graph Models* describes the ERGM model in a brief while also emphasizing the novelty of the algorithm in this context. In Section titled *Data and Analysis Strategies*, we describe the dataset used for analysis and the methodology employed. In the section titled *Results and Analysis*, we present the results and analyses of our experiments and finally conclude the paper in section *Conclusions*.

## Related works

The literature on patent data analysis is vast and varied. It is, thus, nearly impossible to list all the important works in conjunction patent analysis which deal with citations and other bibliometric measures. In this section, first we state a few important works in that regard which are relevant in context to our work in the broader sense. Also, we devote a section to the relevant works that involve ERGMs, which again is a vast research landscape on its own.

### Patent analysis

It has already been established that statistical analysis of international patent documents acts as an invaluable instrument for technological planning and analysis within companies. Patents are a known source of detailed information, providing comprehensive coverage of technologies and countries, a relatively standardized level of invention, and a long time-series of data [17]. So, it essentially provides us with a technological indicator to measure technological growth, which in turn could be extrapolated to get a better understanding of the relation and mutual dependence of innovation and economics [18, 19].

The field of quantitative evaluation of scientific impact is built upon the intrinsic notion that the scientific standard of papers [20], scholars [7, 21], journals [22], universities [23] and countries [24] can be measured by metrics based on the citations received. Bibliometrics has been employed in a variety of scenarios to measure and analyze citations since they provide a rich source of information. Scientific papers and scholarly articles have been investigated using various bibliometric tools, especially citations for a long period [25]. While it is nearly impossible to study the characteristics of the complete citation graph of scholarly articles, researchers have chosen to focus on either different aspects of the network or on a subset of the graph [26]. Patent citation analysis gained traction relatively late (in the 1990s) compared to their scholarly articles’ counterpart. One of the early studies to measure the technological impact based on patent citations was done by Karki [27], who proposed several technological indicators based on citations among patents. Criscuolo *et al.* [28] have investigated the significance of R&D internationalization with respect to host country innovation systems providing aids in quantifying relative asset augmentation compared to the exploitative nature of foreign-located R&D Some studies, like the one by Albert *et al*. [29], have considered only citations counts as indicators of industrially important patents. The pattern of knowledge flows, as indicated by patent citations between European regions, has been studied by Maurseth and Verspagen [30]. The authors observed that patent citations are industry-specific and citation propensity increases between geographical regions that are focused on industrial sectors with specific technological linkages between them. It has also been observed that the frequency of patent citations is high between regions which belong to the same linguistic groups.

Almeida [31] investigated the contribution patterns of multinational firms in the U.S. semiconductor industry through citation analysis. The study reported that foreign firms also contribute to local technological progress significantly. In a study, Hall *et al.* [32] found that firm market value, as indicated by the *Tobin’s Q ratio*, was correlated to the citation-weighted patent portfolio of the firms. Carpenter *et al*. [33] and Fontana *et al.* [34] juxtaposed award-winning inventions in the form of patents against patents belonging to a control group, demonstrating that *important* patents are more cited. In fact, it was found that the average number of citations received by important patents was about 50% higher than other patents. Zhang *et al.* [35] proposed to weigh 11 indicators of patent’s technological value by using Shannon entropy and selected forward citations. Thus, patent analysis spans a multitude of research areas right from patent search [36], patent classification [37, 38] and categorization to measuring the social impact of patents [39, 40]. Patent citation analysis can thus act as a bridge between these overlapping areas while providing a cursory overview of the patent landscape. The primary reason for using citations received as a quality indicator is that citations are able to capture some form of knowledge spillovers. In fact, citations either serve a similar role or allows building new technology from an existing one [41]. Consequently, citation chains are helpful in tracing technological evolution. In this regard, the centrality of patents in the citation network can be used to assign scores to patents. However, not all measures of centrality are equally applicable in all scenarios. There are situations for instance, where we would like to quantify the citations received as positive but not necessarily how many citations are spawned. Also, there are various challenges and limitations to citation analysis of patents, including lack of technical knowledge to process patent citations, geographical constraints and language barriers [42]. Some problems and critiques to citation analysis are presented in the papers by Fortunato *et al.* [43], MacRoberts and MacRoberts [44] and Garfield *et al.*[45]. For patents as well, citations often are used as proxies or indicators of knowledge growth and spillovers. However, as pointed out by Jaffe *et al.*[41], this does come with some inherent limitations especially when studying the mechanisms associated with the movement of knowledge flows. Our research also operates under these set of assumptions. There have been prior studies on patent collaboration network [46] for specific fields, but none have focused on the patent citation network from the perspective of categorical sectors (explained later). A number of studies [13, 47, 48] have focused on the sociological aspects of a patent citation network. In particular, Agrawal *et al.*[47] observed that knowledge flows to an inventor’s prior location are approximately 50% greater than if they had never lived there, suggesting that social relationships, not just physical proximity, are important for determining knowledge flow patterns. While such sociological factors are equally relevant for our study, it is often difficult to replicate similar findings for other datasets due to difference in patenting processes. The impact of innovation on revenue generation for renowned companies has been studied by Singh *et al.*[49]. Recently Kuhn *et al.* argued that due to systemic changes in the data generation process, many of assumptions in patent citations are no longer valid [50].

In this paper, apart from presenting detailed descriptive analyses of the citation networks, we also use ERGMs to study how technical features of the patents and social processes influence citation formations. Similar to the work done by An and Ding [51], we model the effects of a list of covariates we extracted from patents distinguishing between receiving and sending citations. In this study, we provide theoretical expectations on the effects of the covariates and discuss how different patent characteristics can matter for citations. We also aim to account for the homophily in citation formations, especially with respect to the same country and the same language.

### Exponential random graphs

Exponential Random Graph Models (ERGMs) are statistical models of network structure, permitting inferences about how network ties are patterned [52]. ERGMs have been applied to several fields such as economics [53], sociology [54], political sciences [55], international relations [56], medicine [57] and public health [58] with varied application ranging from modeling micro-blog networks [59], studying relational coordination among healthcare organizations [60] to strategic management research [61]. Social network models too have attracted considerable attention from physicists [62, 63] and have been pivotal in the development of interdisciplinary perspectives [64]. On the other hand, networks have been extensively studied from the perspective of preferential attachments. For instance, Barabási *et al.* [65] demonstrated that in a large network such as World Wide Web, despite its apparent random character, the topology of the graph has a number of universal scale-free characteristics. While Jeong *et al.* [66] proved that nodes acquire links depending on the node’s degree, offering direct quantitative support for the presence of preferential attachment in scientific citation networks. However, little research exists on studying and understanding patent citation networks from a social and structural perspective. We believe our current work will help researchers gain a preliminary understanding of how treating patent citation networks can lead to a more inclusive and interdisciplinary understanding of the impact of patents.

## Exponential random graph models

We use Exponential Random Graph models (ERGMs) to examine citation patterns. ERGMs are a well established family of statistical models for the analysis of network data. An ERGM assumes that the observed network is a random network, and comes from an exponential family of probability distributions [67, 68]. Let us assume that *x* is a network with *N* nodes. Mathematically, it can be represented by a *N* × *N* matrix *x* = [*x*_*i*,*j*_] of binary tie variables. Here *x*_*i*,*j*_ = 1 if patent i cites patent j, otherwise *x*_*i*,*j*_ = 0. ERGM may be written in the following form:

$$\begin{array}{c} \pi \left(x\right)=\exp(\sum_{s=1}^{S}\theta_{s}g_{s}\left(x\right))/Z \end{array}$$ (1)

where *π*(*x*) is a probability of network *x* and *Z* is a normalizing constant to ensure that this probability distribution sums to 1; *θ*_*i*_ are model parameters, and *g*_*s*_(*x*) are the network statistics that will be employed for the study. The number of statistics *S* may be large, but as it states in Eq (1) we have one parameter for one statistic. The value *g*_*s*_(*x*) may be defined for any feature of interest. A simple example is the number of network ties *g*_*L*_(), which is given by:

$$\begin{array}{c} g_{L}\left(x\right)=\sum x_{i,j} \end{array}$$ (2)

The typical network features are transitivity, degree distribution and homophily [16]. Statistics for these structural features were proposed by Snijders *et al.* [69]. ERGMs have been known to account for both endogenous network formation processes and covariate effects. ERGMs can be used to model many different network features simultaneously and do not assume that these features are isolated. In order to find a feature in the network data or to check a hypothesis, researchers may compare the value of statistics of the network under study with that in a random network. However, real networks are not random, and instead of comparing them with completely random networks it is better to compare with a NULL model, that takes into account both transitivity, degree distributions, and all the other important network features. The capability of ERGMs to model all these features simultaneously solve the problem of the NULL model in an elegant way.

ERGMs permit statistical inference from the observed network *x*_*obs*_. The parameter of the model should be estimated by maximizing the likelihood *π*(*x*_*obs*_). It may be shown [70] that maximizing the likelihood for this probability distribution is equivalent to finding the solution of the following system of equations:

$$\begin{array}{c} g\left(x_{obs}\right)=E_{\theta }g\left(x\right) \end{array}$$ (3)

where *E*_*θ*_ *g*(*x*) = ∑_*x*_ *π*(*x*)*g*(*x*) are expectation under the ERGM distribution, and we adapted the vector notations *g*(*x*) = (*g*_1_(*x*), *g*_2_(*x*),.., *g*_*S*_(*x*)), *θ* = (*θ*_1_, *θ*_2_,.., *θ*_*S*_). In completely random networks, we would have *θ*_*s*_ = 0 for all the statistics, but real networks are not completely random. If the estimated parameter *θ*_*s*_ is significantly larger than zero, then the corresponding statistics *g*_*s*_(*x*_*obs*_) is larger than might be expected by chance given all the other parameters of the model. This statistical methodology allows a detailed analysis of the network data and is particularly suitable for hypothesis testing.

Nevertheless, as has been pointed out by An and Ding [51], studying citation networks using ERGMs poses several challenges. It is a known fact that it is difficult to fit ERGMs on large networks. For large networks composed of thousands of nodes and edges such as the ones studied in this paper, fitting ERGMs may be very slow. This is owing to the fact that ERGMs rely on Markov chain Monte Carlo to simulate networks for estimations. The estimation of parameters of ERGMs by maximization of the likelihood is a computationally expensive procedure. In practice, the size of the largest network for which ERGM parameters may be estimated by this method is limited to a few thousand nodes [71]. For larger networks, the solution of Eq (3) cannot be found in a reasonable time. Researchers either have to study smaller sub-networks or to use very crude approximations, like for example, contrastive divergence or pseudolikelihood [51, 71]. Recently a very efficient algorithm for the solution of Eq (3) was proposed [72, 73]. This new algorithm may be considered as a modification of the algorithm proposed by Laurent Younes [74] and was implemented in open-source software (available at: https://github.com/stivalaa/EstimNetDirected) for fitting ERGMs to large directed networks [75]. In this paper we have used this software for the analysis of patent citation networks. Loading data and carrying out computations with large networks demands a lot of memory, and we use a cluster of computers to solve this issue.

A citation network is a directed network, and to analyze it we adapt statistics which are typical for such networks. We model both the endogenous structural features and the effect of the patent attributes. The transitivity is modelled by AltKTriangleT (GWESP), the degree distributions are modelled by AltInStars (GWIDEGREE) and AltOutStars (GWODEGREE), and the two-paths are measures by AltTwoPathesTD (GWDSP) [16, 76]. Besides, we studied the effects of some attributes on the network formation, such as the sender and the receiver effects. The sender effect is the effect of binary patent attributes *a*_*i*_ on the probability of tie *x*_*i*,*j*_ which may be studied by adapting the following statistic:

$$\begin{array}{c} g_{send}\left(x\right)=\sum_{i,j}a_{i}x_{i,j} \end{array}$$ (4)

while the receiver effect may be studied by

$$\begin{array}{c} g_{rec}\left(x\right)=\sum_{i,j}a_{j}x_{i,j} \end{array}$$ (5)

The homophily effect increases the probability of ties between node *i* and *j* if these nodes share the same value of an attribute:

$$\begin{array}{c} g_{homo}\left(x\right)=\sum_{i,j}\delta_{a_{i},a_{j}}x_{i,j} \end{array}$$ (6)

where *δ* is a Kronecker delta function [75, 77]. The homophily has the same effect on both ties *x*_*i*,*j*_ and *x*_*j*,*i*_. A citation network is a special type of directed network, because a patent only cites patents that are already published. Mathematically this means that the probability of a tie *x*_*i*,*j*_ is very small if *d*_*i*_ < *d*_*j*_, where *d*_*i*_ is the date of publication of patent *i*. One can expect that the patents do not cite patents that are not published yet, and hence probability of citations from *i* to *j* is close to zero if *d*_*i*_ < *d*_*j*_. Otherwise we would have *g*_*date*_(*x*)>0. From Eq (1) one can see that the probability *π*(*x*) of such networks will be very small if the corresponding parameter has a large negative value [51]. We incorporated this effect by adding another statistic

$$\begin{array}{c} g_{date}\left(x\right)=\sum_{i,j}H\left(d_{j}-d_{i}\right)x_{i,j} \end{array}$$ (7)

where *H*(*y*) is a unit step function, defined as:

$$\begin{array}{c} H\left(y\right)=\{\begin{array}{cc} 0, & \text{if}\;y\leq 0\\ 1, & \text{otherwise.}\; \end{array} \end{array}$$ (8)

We know in advance that in citation networks the corresponding parameter value *θ*_*date*_ is negative. Following An and Ding [51], instead of estimating the value of this parameter, we always used a constant value *θ*_*date*_ = −10^10^.

Finally, we fit ERGMs only on the sector citation networks in which isolated nodes (that is, patents that do not cite or get cited by any other patents) are discarded [78]. The results based on the sector networks can be interpreted as capturing citation patterns among the patents from that particular sector. One limitation of this method is that some of the results obtained for sub-networks may not generally be applied to the complete citation network comprising of all the sectors. In general, results become more reliable as the size of sub-networks increases.

## Data and analytical strategies

### Dataset

For our experiments, we worked with the European Patent (EP) sub-collection from the MAtrixware REsearch Collection (MAREC). This sub-collection of patents consisted of around 1.2 million (granted) patents in English, German and French for a period of 32 years (1976-2007), provided in XML format. From each patent document, we extracted the relevant bibliographic and meta-information such as Date, Language, Title, Applicant Country, Applicant, List of Inventors, Classification Codes, Patent-Patent (P-P) citations, and Patent-Non-Patent (P-NP) citations. In this study, we only focus on the patent to patent citations and thus ignore the patent to non-patent citations, such as to scholarly papers, books *etc*. We discarded some patents with missing Applicant Country, Classification Codes and P-P citation fields. The total number of curated patents thus stood at 757,869. For building a citation network from these curated patents, we had to eliminate patent citations outside of European Patent Office (EPO), since we did not have any information about such cited patents (*e.g.,* patents from the National Patent Offices, or the World Intellectual Property Organization (WIPO), *etc.*) apart from EPO patents contained in this sub-collection. Also, we had to eliminate citations that belonged to the time period beyond our collection scope, such as the ones from before 1976. The citations formed by these patents that are out of our dataset and older than 1976 are termed as “non-relevant” (although we recognize that they might be). So, the initial network consisted of 3,252,497 citations, and after eliminating non-relevant citations, the network reduced to 646,537 citations.

European Patents registered with the European Patent Office (EPO) follow the International Patent Classification (IPC) system (https://www.wipo.int/classifications/ipc/ipcpub/) under which each patent can be broadly classified under one (or more) of the eight classes or *sectors* from A to H.

**A**: Human Necessities
**B**: Performing Operations; Transporting
**C**: Chemistry; Metallurgy
**D**: Textiles; Paper
**E**: Fixed Constructions
**F**: Mechanical Engineering; Lighting; Heating; Weapons; Blasting
**G**: Physics
**H**: Electricity

**Fig 1:**
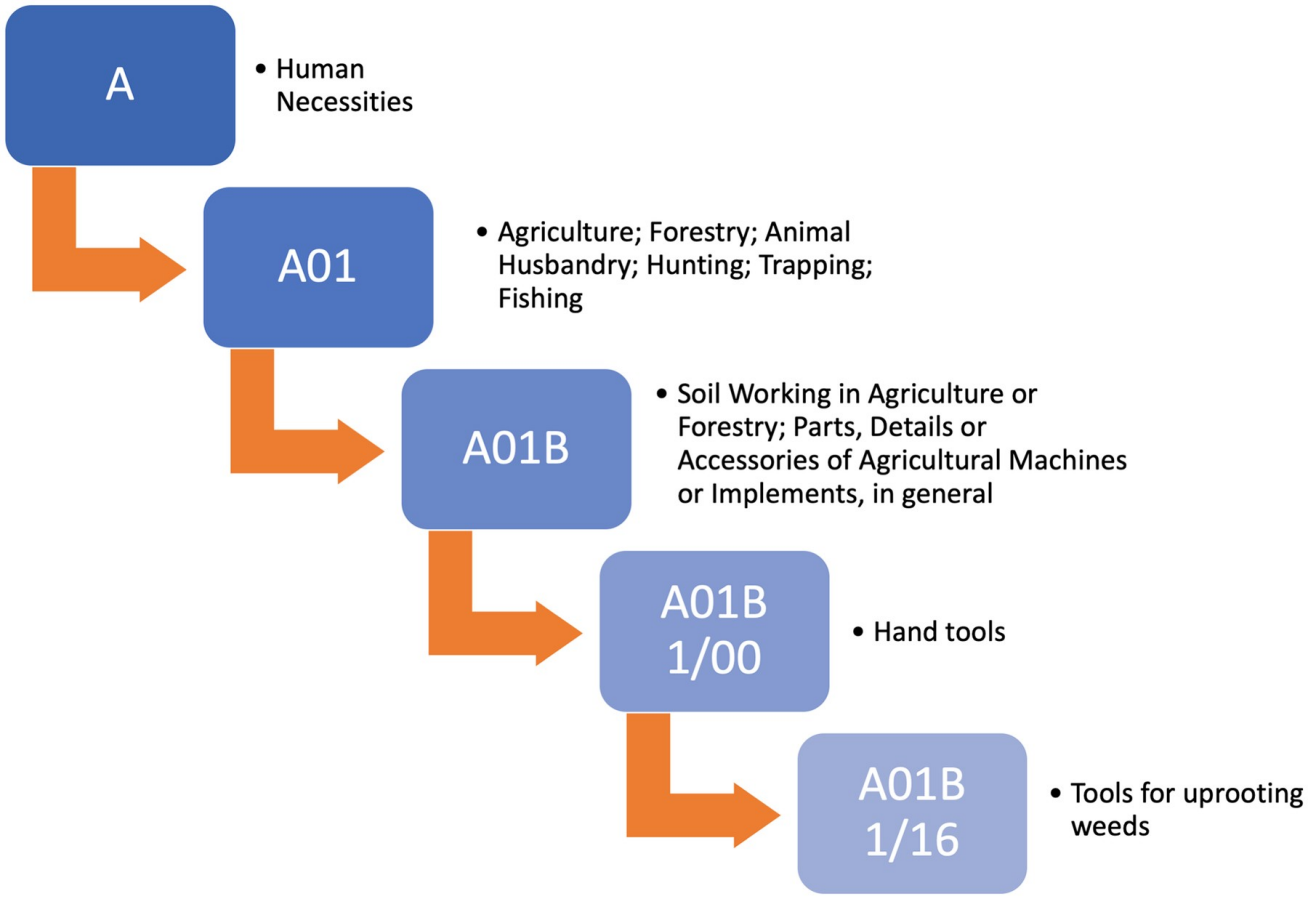
IPC classification example.

Each of these sectors are further classified into four levels of sub-classes or *categories*. Fig 1 describes one instance of such classification.

In Tables 1 and 2, we provide the sector wise and category wise (the third level of the classification hierarchy) distributions of the curated patents respectively. One can notice that the sum of the number of patents in Table 1 is more than 757,869. This is owing to the fact that a single patent can belong to multiple sectors (and categories). In other words, when we counted the number of patents belonging to a particular sector (or category), we considered all the patents that were classified with the sector (or category) label. For instance, in the dataset, patent document EP0000001 has been classified with sector labels B, F, G and H. Thus, while counting for the number of patents for sectors B, F, G and H, we consider EP0000001 to appear in each one of them.

**Table 1:** Sector wise distribution of patents.

Sector	#Patents
B	218000
C	183809
G	167318
H	157790
A	151226
F	97498
E	33628
D	23581

**Table 2:** Category wise distribution of patents (top 10).

Category	#Patents
A61K	47884
C07D	28247
A61P	27234
H01L	24857
C07C	24158
G01N	23894
G06F	23175
B01J	19941
H04L	19807
A61B	18887

In this paper, we study the citation networks built from sectors B and E. Our objective is to try and compare the results of our experiments on different sized networks. The corresponding networks of sector B and E have 101,128 and 12,256 citations, respectively. The networks for sector B and E have been made available for public-use on https://github.com/Manajit89/ERGM-patent-analysis. For building these citation networks, we retained citations from the original network where both the citing and cited patent belongs to sector B or E. Since sector B has the largest share of patents, we expect that there will be significant differences of such a large network when compared to a relatively small network such as the one built from patents in sector E.

### Analytical strategies

In the literature, scientific collaborations and structure of science have been studied using citation network methods [79–81]. Particularly, citation networks have been examined to study information diffusion [82, 83] and scholarly impact [84–86]. Most of these previous works on studying citation networks have been focused on simply providing descriptive analyses. The work done by An and Ding [51] was one of the first studies that looked beyond descriptive analyses. ERGMs were employed to investigate how citation formations are affected by technical features of the scientific publications and social processes.

To the best of our knowledge, no such study exists with respect to patent citation networks. Taking a cue from the work by An and Ding [51], we extracted a host of covariates from patent documents (see later) and model their effects, distinguishing effects on receiver and sender characteristics of citations. We also provide theoretical expectations on the effects of the covariates and demonstrate the influence of patent characteristics on patent citation formation. The analyses presented in this paper also aims to account for the homophilous nature of the citation formations [87]. We anticipate that citations have a tendency to form between patents that are in the same language and patents that belong to the same country.

Further, we also analyze multiple endogenous network formation processes in citations. We explore citation transitivity (*i.e.,* if Y is cited by X and Z is cited by Y, then Z is more likely to be cited by X). We posit that transitivity in citations can occur because inventors (and sometimes even patent examiners) may use a *snowball strategy*[51] to find new patents and similar documents by following the references of other such documents. We also examine *preferential attachment* [51] or advantage of citation cumulation, namely, the fact that patents that have historically received a higher number of citations are prone to receive even more citations over time. We anticipate that some patents will receive more citations than other patents, and hence the number of citations received by different patents will vary widely. While at the same time, we expect that the variation in the outgoing citations might be small. Moreover, our analysis takes into account a distinctive feature for citations *i.e.,* there are no forward referencing in patents, implying that earlier patents cannot cite later ones.

For each patent, we extracted and constructed a series of covariates. These covariates are employed to model a host of possible mechanisms for the formation of citations. For example, a patent may cite another patent because they are written in the same language [88] or if they belong to several categories indicating a diverse patent. To clarify, assuming two patents on similar technologies (one in English and other in German) that could be cited by an applicant, they tend to lean towards the reference patent written in the language of the citing patent. A recent patent may invoke interest in some sectors or categories, and this might result in accruing more citations and so on. As described below, each of these citation mechanisms can be quantified and compared using various covariates, and different aspects of the mechanisms may be measured by each of the covariates. Thus, it is hard to differentiate between the mechanisms and the effect of the covariates to the mechanisms.

**Fig 2:**
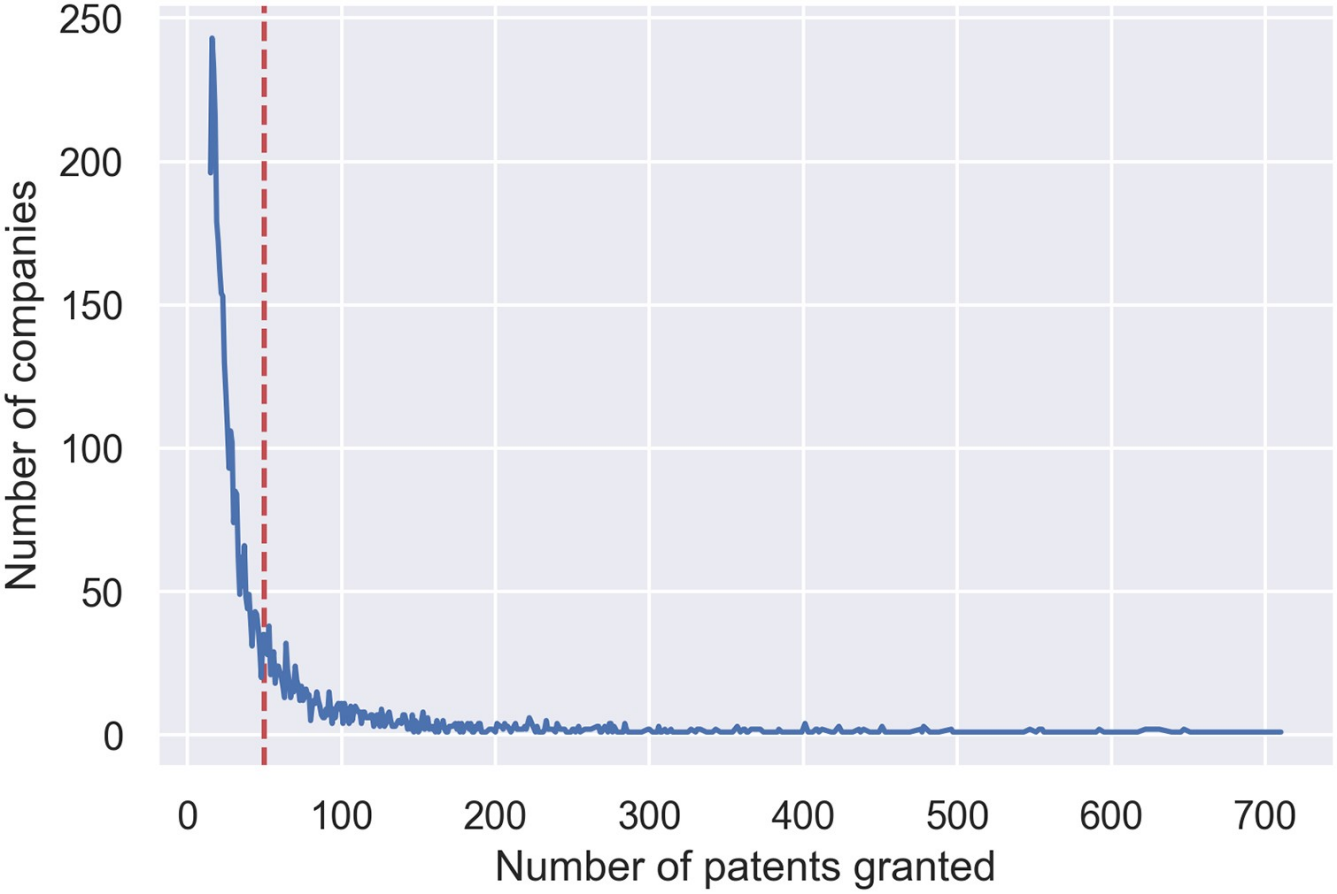
Patent distribution by top-5000 companies (best viewed in color) without top-100 companies. The threshold line for *Prolific Applicants* is marked with a red dashed line.

The patent covariates that we studied in this paper are listed below:

1. **Swiss Patent** (binary variable, IsSwiss = 1; others = 0): We wanted to study the effect of country of origin of the patent on citation formations. In particular, since this is a study funded by a Swiss National Science Foundation (SNF) project, we were interested in observing the characteristics of the patents when they are filed by applicants belonging to Switzerland. Obviously, we could repeat the study focusing on any other country.
2. **Patent Recency** (binary variable, IsRecent = 1; 0 otherwise): We expect that how long ago a patent has been granted will affect how many citations it attracts. For this, we define *recency* as a measure which can be of either five or ten-year category. This implies that the patent was granted in the last five or ten years, respectively. Recency effect in the network, especially of scientific citations, already studied in the literature [89], showed that sometimes citing a recent scientific can amount to both reputation and visibility of the cited article over a longer period. In lieu of that, we also wanted to confirm if a similar hypothesis holds for patent citation networks.
3. Whether the patent belongs to **Multiple Categories** (binary variable, IsTrue = 1, 0 otherwise): This parameter takes into account the diversity of a patent. If a patent belongs to four or more categories (as defined in Section Dataset), it is considered as a multi-categorized patent. We expect that patents with more categories will attract more citations by virtue of belonging to several different domains.
4. Whether the patent was filed by a **Prolific Applicant** (binary variable, IsTrue = 1, 0 otherwise): We wanted to observe the effect of a patent when it is produced by a company or an organization which has a history of filing a large number of patents (*i.e.,* a prolific company). This definition is supported by Fig 2, where we show a truncated view (of 5000 companies from Table 4 except the top 100) of the number of companies with the highest number of patent grants within the period 1976-2008. We considered the companies with at least 50 granted patents within the mentioned time period as ‘Prolific Applicant’.
5. **Language** (binary variable): We expect that the language that a patent is filed in will also affect how citations are modelled. In particular, we study the effect of this parameter with respect to each of the three languages in the dataset, English (EN), German (DE), and French (FR).

Statistics that are exclusively based on the network information shared by node pairs, *a* and *b*, also known as *dyads* are referred to as dyadic endogenous network statistics. We create three dyadic variables to represent assortative mixing mechanisms in citation formations.

- Whether any two patents belong to the same country *i.e.,* they are produced by applicants from the same country (yes = 1; no = 0).
- Whether any two citing patents are written in the same language (yes = 1; no = 0).
- Whether there is an overlap between the categories of any two patents (yes = 1; no = 0), *i.e.,* the two patents share some of the common categories.

We employ two methods to analyze the citation data. Firstly, we present descriptive analyses of the citation network in patents. We present the distribution of the number of patents over the years, the most cited patents, the top companies, *etc.* Also, we describe the primary characteristics of the citation network, including:

- transitivity (the propensity of a patent to cite the references of references)
  $$\begin{array}{c} T=\frac{3\times \text{number}\;\text{of}\;\text{triangles}\;\text{in}\;\text{the}\;\text{network}}{\text{number}\;\text{of}\;\text{connected}\;\text{triples}\;\text{of}\;\text{nodes}\;\text{in}\;\text{the}\;\text{network}} \end{array}$$ (9)
- density (the fraction of all possible citations that are present in the observed citation network)
  $$\begin{array}{c} D=\frac{\left|E\right|}{\left|V|(|V|-1\right)} \end{array}$$ (10)
  where |*E*| and |*V*| represents the total number of edges and vertices (nodes) in the network.
- centralization (the propensity of citations to asymmetrically converge on a few patents),
- indegree (the number of citations received),
- outdegree (the number of the patent cited other patents),
- betweenness (the number of times a patent is on the shortest path that connects any two other patents).
  $$\begin{array}{c} g\left(v\right)=\sum_{s\neq v\neq t}=\frac{\sigma_{st}\left(v\right)}{\sigma_{st}} \end{array}$$ (11)
  where *σ*_*st*_ is the total number of shortest paths from patent *s* to patent *t* and *σ*_*st*_(*v*) is the number of those paths that pass through *v*.

In the context of patents, indegree may be perceived as indicative of a patent’s influence in the field, outdegree can be an indicator for a patent’s interaction with other patents, and betweenness as reflective of a patent’s brokerage power (that is, the concerned patent plays the role of connecting diverse topics and sub-categories).

By receiver effects, we aim to capture the likelihood of patents with certain traits to overtly cite patents with similar traits than citing patents without those traits. Similarly, the sender effects capture the likelihood of patents that are more likely to be cited by patents with certain traits rather than those without those traits. The assortativity of the citation mechanisms is measured by homophily effects and indicate whether patents with the same characteristics (*e.g.,* belonging to the same category or geographical location) are more inclined to cite one another than those with different characteristics. We fit this model on the sector citation networks.

Additionally, we also incorporate multiple endogenous network formation processes and a variable which indicates forward referencing. We have included the geometrically weighted edgewise shared partners (AltKTriangleT) to account for transitivity in the citations. AltKTriangleT indicates the propensity of citations to form a triangle (that is, if X cites Y and Y cites Z, then X is expected to cite Z). AltTwoPaths represents the propensity of citations to run across two paths but not form a triangle (that is, X cites Y and Y cites Z, but X does not cite Z). Generally, if there is a positive coefficient for AltKTriangleT and a negative coefficient accompanies it for AltTwoPaths, it indicates that higher levels of transitivity are present in the citations [90, 91].

## Results and analysis

### Descriptive statistics

**Fig 3:**
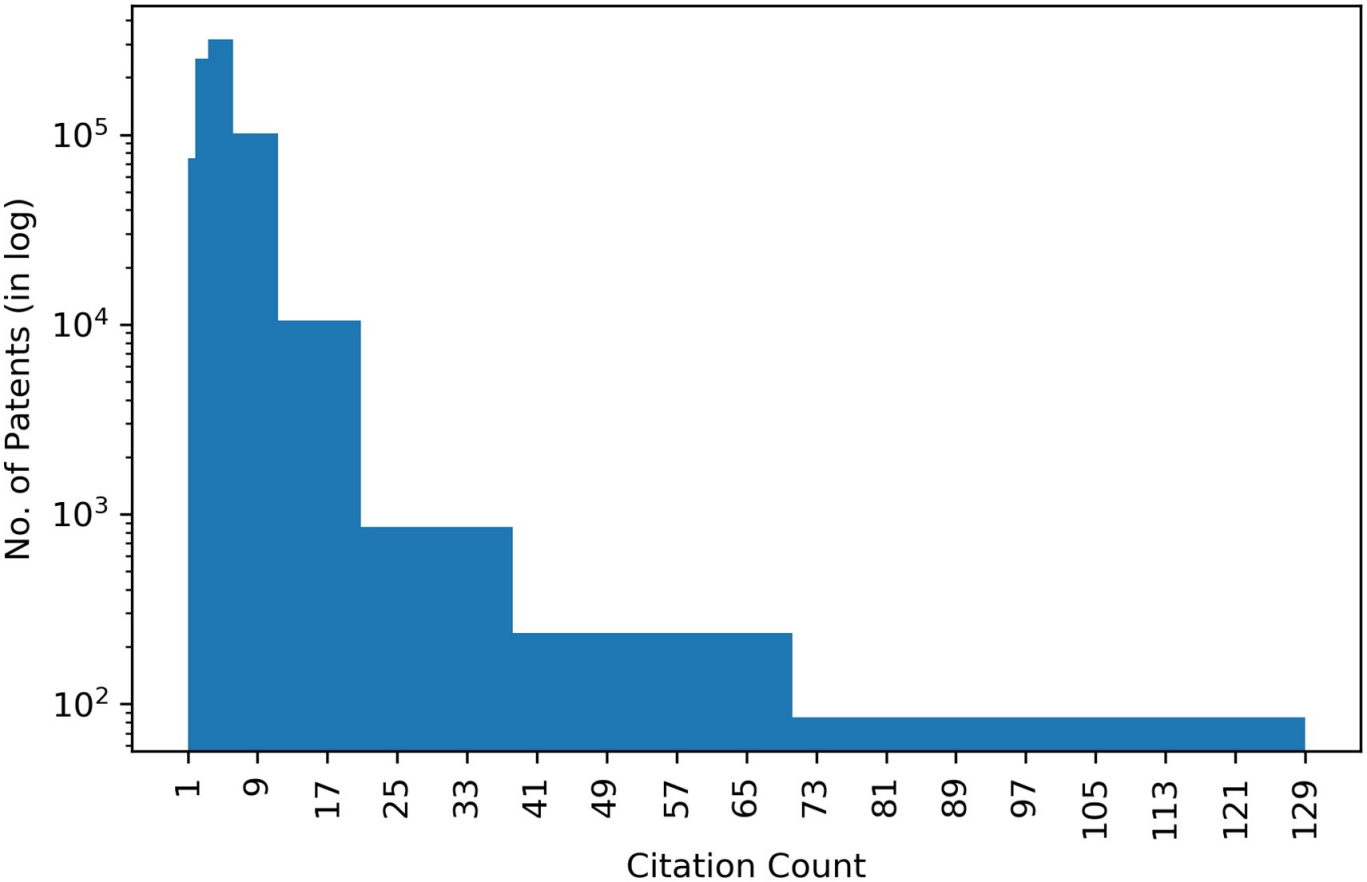
Patent citation count distribution.

We begin by presenting some statistics about the patent citation network. Table 3 presents patents with the highest number of citations. The citation distribution for the complete network is provided in Fig 3. From this figure, we can observe that only few patents have a high count of citations, while the majority of the patents receive very few citations.

**Table 3:** Top-5 patents by citation count.

PatentID	#Citations
EP-1652580	129
EP-1829684	129
EP-1621338	127
EP-1635216	126
EP-1757984	126

In Table 4, we list the highly influential companies or organisations with the highest number of granted patents within the aforementioned time period. We did not need to perform any form of name normalisation since in the dataset there were no discrepancies associated with applicant names. In this table only companies with 10 or more patents are presented, which we label as ‘Prolific Applicant’ and used as covariate in Section “Analytical Strategies”. Table 5, on the other hand, describes the country-wise distribution of patents. It is interesting to note that even though the dataset is concerned with patents filed at the European Patent Office, most of the patents originate from the United States of America (U.S.). A summary of the descriptive statistics of the complete citation network is presented in Table 6. As indicated by the extremely low density, the network is extremely sparse. Low centralization score also depicts that the citations are not concentrated only on a few patents. This is in agreement from the findings of An and Ding [51] with respect to academic citations. The degree of reciprocity is also very low which indicates that there are only about 1% mutual citations, due to lack of forward references. A low degree of transitivity—about 5% of the patents cite their references’ references is also noted. The reciprocity in the network arises due to some discrepancies in the dataset, *i.e.,* due to presence of some cycles within the network.

**Table 4:** Patent distribution for companies/organizations.

No.	Company/Organization	#Patents
1	SIEMENS AG	11939
2	MATSUSHITA ELECTRIC IND CO LTD	5832
3	BAYER AG	5313
4	BASF AG	4575
5	IBM	4328
6	SONY CORP	3927
7	PHILIPS NV	3727
8	GEN ELECTRIC	3702
9	EASTMAN KODAK CO	3691
10	CANON KK	3584
11	BOSCH GMBH ROBERT	3458
12	HOECHST AG	3437
13	CIT ALCATEL	3317
14	SAMSUNG ELECTRONICS CO LTD	3189
15	OREAL	3057
16	HITACHI LTD	2773
17	PROCTER & GAMBLE	2668
18	FUJITSU LTD	2568
19	TOKYO SHIBAURA ELECTRIC CO	2547
20	CIBA GEIGY AG	2468
…	…	
7587	SCHIFFER FA M & C	10
7588	WALKER ASSET MANAGEMENT LTD	10
7589	MASONITE CORP	10
7590	OLIVEIRA & IRMAO SA	10
7591	DEHN & SOEHNE	10
…	…	

**Table 5:** Patent distribution by country (Top-20).

Country Code	#Patents
US	181914
DE	146628
JP	144117
FR	85667
CH	34167
IT	30769
GB	28279
NL	24545
BE	9693
KR	9250
SE	9206
AT	7159
AU	7014
CA	5884
ES	4054
FI	4038
TW	3865
DK	2997
IL	2513
CN	2460
…	…

**Table 6:** Summary statistics of citation network.

Parameter	Value
Density	1.568e-06
Transitivity	0.005
Reciprocity	0.001
Avg. In-Degree	1.1316
Avg. Out-Degree	1.1316
Betweeness Centrality	8.292e-06

We can observe that each patent receives about 0.85 citations. There is a high correlation between indegree and betweenness, suggesting the likelihood that patents that are cited across different categories are also the ones with a higher number of citations. There is also a strong correlation between indegree and outdegree, indicating the tendency that highly cited patents often cite more patents.

**Fig 4:**
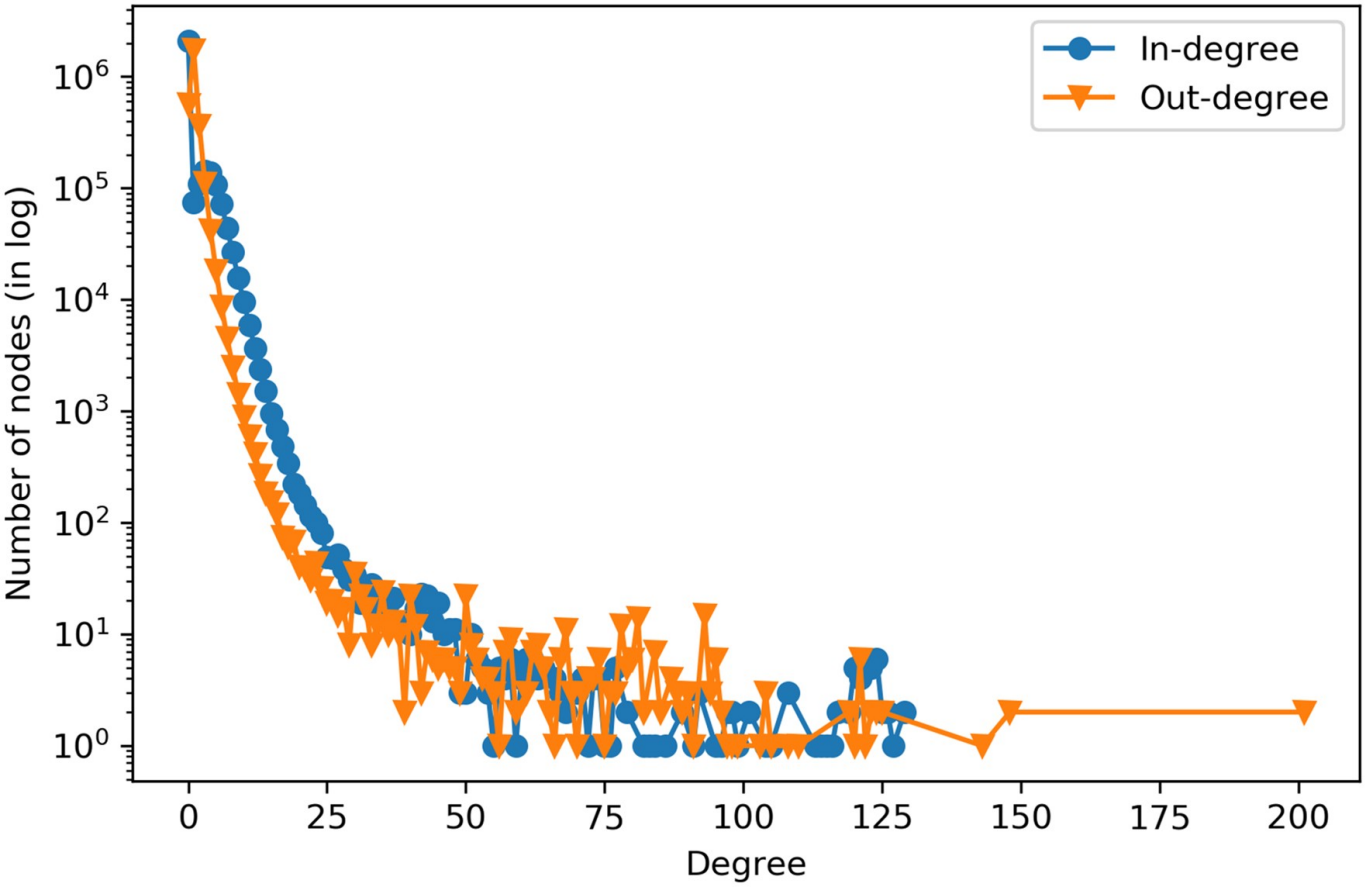
Degree distribution of citations (best viewed in color).

In Fig 4 presents the degree distribution of the full citation network. Both indegree and outdegree show similar patterns with the majority of the patents having both high indegree and outdegree.

**Fig 5:**
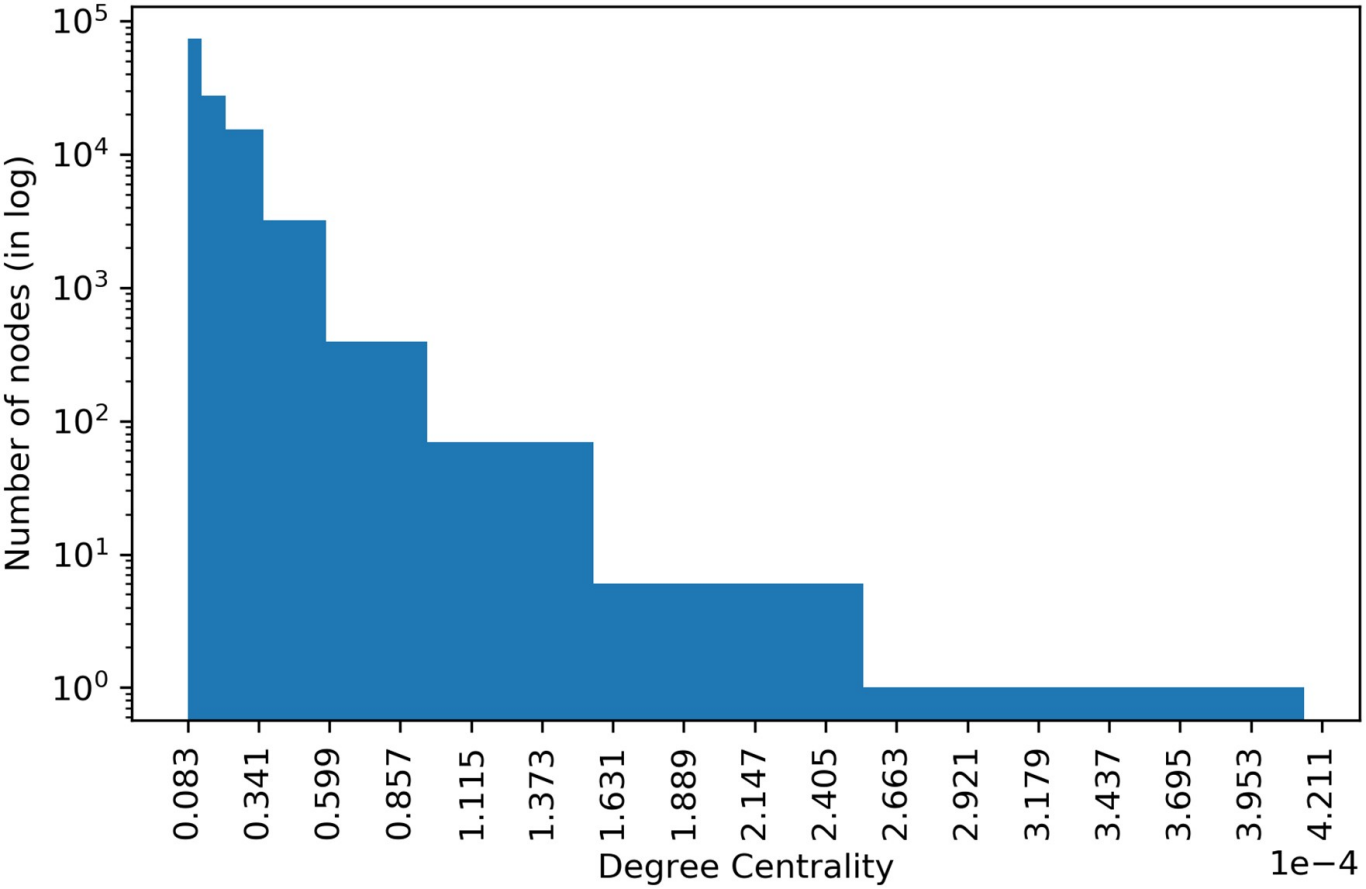
Distribution of degree centrality.

Fig 5, shows the distribution of degree centrality of all patents, and we can observe that a large number of nodes appear with low centrality. In contrast, only a few nodes have high centrality.

### ERGMs

Endogenous network formation processes contribute significantly in citation formations and network inference in general and should be taken into account to improve the quality of inference. A good network generating model should fit density, transitivity, and degree distribution in addition to other effects, that researchers want to study. For computational purposes, we chose to work with sector-based citation network, where the citations within the sectors are preserved, instead of the complete network. In Table 7 we present the covariate effects when the ERGM is fitted on the sector E citation network. “Edges” (arcs) act like regression intercepts and are required to fit the density of the network under study.

**Table 7:** ERGM results for predicting the sector E citation network.

	Estimation	Standard Error
Edges	-9.660834	0.1017339*
*Receiver Effects*		
Swiss Patent	0.268999	0.0200916*
Recency (5 year)	0.06767537	0.01348582*
Recency (10 year)	0.2858105	0.01691144*
Multi-Categorization	-0.5059612	0.01740204*
Prolific Organization/Company	0.130446	0.01019256*
Language (EN)	0.1347897	0.1020575
Language (DE)	-0.118628	0.09924686
Language (FR)	0.1186651	0.1016732
*Sender Effects*		
Swiss Patent	0.1379004	0.01411045*
Recency (5 year)	-0.1649588	0.01139841*
Recency (10 year)	-0.3704575	0.01555657*
Multi-Categorization	-0.07931266	0.00865454*
Prolific Organization/Company	0.06954835	0.009215311*
*Homophily*		
Same Country	1.120555	0.02561618 *
Same Language	0.3065091	0.01443439*
Overlapping Categorization	5.713291	0.02372079*
*Network Structures*		
AltInStars	-1.197857	0.01680285*
AltOutStars	-0.1323321	0.006557173*
AltKTrianglesT	5.095065	0.201787*
AltTwoPathsTD	-2.482252	0.02188758*

Statistical significance (*p* < 0.05) is marked by an asterisk (*).

A large coefficient for “AltKTriangleT” implies that a reference’s reference is more likely to be cited. The negative coefficient for “AltTwoPathesTD” indicates that citations that do not form a triangle have fewer chances of occurring. Observing “AltKTriangleT” and “AltTwoPathsTD” together corroborates that citations have a tendency to be transitive, which could be due to the fact that inventors, generally, tend to snowball-sample the literature to discover and learn about existing technologies within the sector. The negative coefficient for “AltInStars” implies that comparing to other effects, there is no strong preferential attachment.

From Table 7 one can see that besides transitivity another strong effect is the overlapping category. The high positive value of the corresponding parameter means that when two patents share the same category, they are about 300 times (*e*^5.71^ ≈ 300) more likely to cite each other. Also, if the patents were issued in the same country, they are much more likely to cite each other. In fact, a patent generated from a particular country has 3 times more chance of citing a patent from that same country than others. This finding is in line with the research carried out by Singh and Marx [14] where they showed that there is a 77% greater likelihood of within-country knowledge flow in the U.S. than across national borders. While this would seem derivative, it was particularly interesting to see the same effect in a different dataset using a robust approach like ERGMs. Combining the above two observations, likelihood of patents being cited because they belong to same categories and that they are generated from country, also indirectly verifies the findings of Agrawal *et al.* [13] where they pointed out that both geographical and social proximity have a positive influence on patent citations.

We also observe a tendency to cite patents written in the same language. However, comparing to “same country” and “overlapping category” the effect of the patent language on citation pattern is not very strong. In literature, there have been studies focused on whether ethnicity has a role to play in information diffusion [11]. For instance, Kerr [12] compared the knowledge production and diffusion between two different ethnic communities in the U.S. and showed that poor access to the codified and tacit knowledge regarding new innovations does contribute to slow technology diffusion. While very relevant to our study, verifying if the same holds true for our context was out-of-scope since we did not have information regarding the ethnicity of the inventors of patents in the MAREC dataset.

Often it is desirable to identify an important patent as early as possible. A simple measure of the value of a patent is the number of citation the patent receives. However, the number of citations depends on both time and many other factors that we want to study. We will measure these effects by “receiver effect”. The corresponding parameter values in Table 7 measure the effect of different covariates on the patent value. It is intuitive that, in general, recent patents will have fewer citations than older ones. However, our results clearly demonstrate (see Receiver Recency effects in Table 7) that more recent patents are more likely to be cited. This could be attributed to the fact that often newer patents provide incremental updates on an existing patented technology, and hence they do not necessarily cite older patents.

Currently, a trend toward interdisciplinary research is observed in science. To the best of our knowledge by now, nobody studied the impact of interdisciplinarity on the patent value. We can consider the patent as interdisciplinary if it has many categories in its classification. We cannot predict it intuitively, but the results from Table 7 suggest that interdisciplinarity does not increase the patent value. In general, patents with fewer categories are more likely to be cited.

Not surprisingly, the patents from top companies have higher value and are more likely to be cited, and this is confirmed by our results. Besides, our results clearly show that on average Swiss patents are more likely to be cited.

Table 8 presents the ERGM model fitted on the Sector B citation network. The number of patents (nodes) in this network is almost 7 times more than that of in Sector E. However the results presented in Tables 7 and 8 are qualitatively the same. It should be noted that when while analyzing the citation networks of each sector with ERGMs, we operate under the assumption that each sector citation network is independent of each other.

**Table 8:** ERGM results for predicting the sector B citation network.

	Estimation	Standard Error
Edges	-13.1524	0.2792836*
*Receiver Effects*		
Swiss Patent	0.3411597	0.03394782*
Recency (5 year)	0.202162	0.03496354*
Recency (10 year)	0.5098135	0.03048308*
Multi-Categorization	-0.6633106	0.03248871*
Prolific Organization/Company	-0.004699791	0.009330533
Language (EN)	1.214152	0.2773483*
Language (DE)	1.050985	0.2756068*
Language (FR)	1.277216	0.2796773*
*Sender Effects*		
Swiss Patent	0.2736611	0.03021695*
Recency (5 year)	-0.351124	0.02299567*
Recency (10 year)	-0.4450601	0.02443803*
Multi-Categorization	-0.402847	0.02749438*
Prolific Organization/Company	-0.04666712	0.01378666*
*Homophily*		
Same Country	1.247913	0.03255275*
Same Language	0.1931007	0.02258632*
Overlapping Categorization	6.460526	0.05387754*
*Network Structures*		
AltInStars	-0.801325	0.02246942*
AltOutStars	-0.2367434	0.01797419*
AltKTrianglesT	6.670764	0.5784175*
AltTwoPathsTD	-1.799021	0.03105744*

Statistical significance (*p* < 0.05) is marked by an asterisk (*).

### Analysis of citation network among top companies

Often in a large network, we can witness the “small-world” effect where certain nodes can be reached within a few hops of each other. This effect has been studied extensively in the literature [92, 93]. Bialonski *et al.* [94] showed that small-world characteristics of interaction networks occur due to the spatial sampling of dynamical systems. Ansmann and Lehnertz [95], proposed the use of *surrogate networks* which preserves the strength of the full network in order to study the network characteristics. Surrogate networks also facilitates the presence of small-world, in that respect, which sometimes can provide additional information about network-specific characteristics and thus aid in their interpretation.

**Fig 6:**
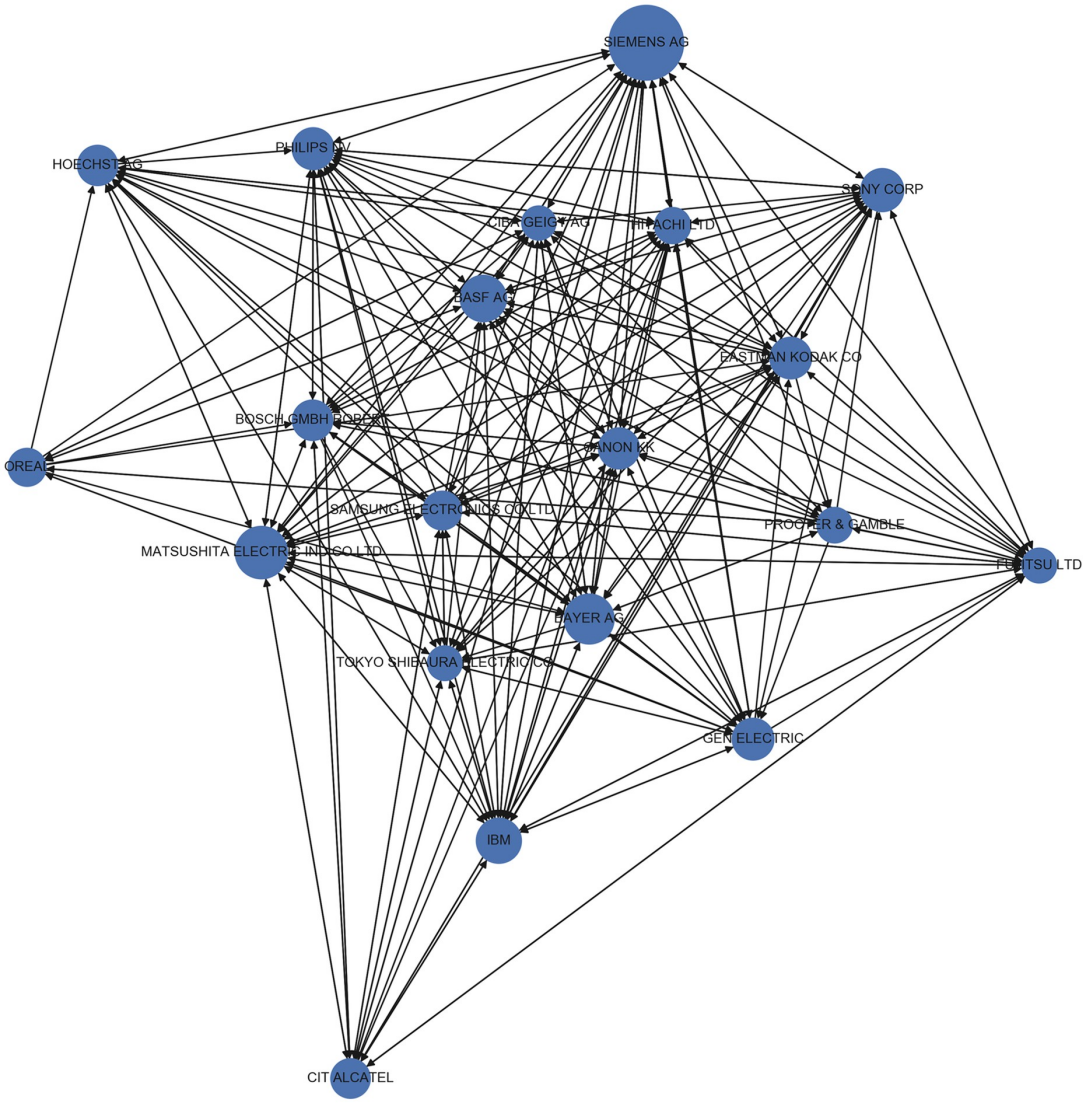
Citation network among top-20 prolific companies (best viewed in color).

**Fig 7:**
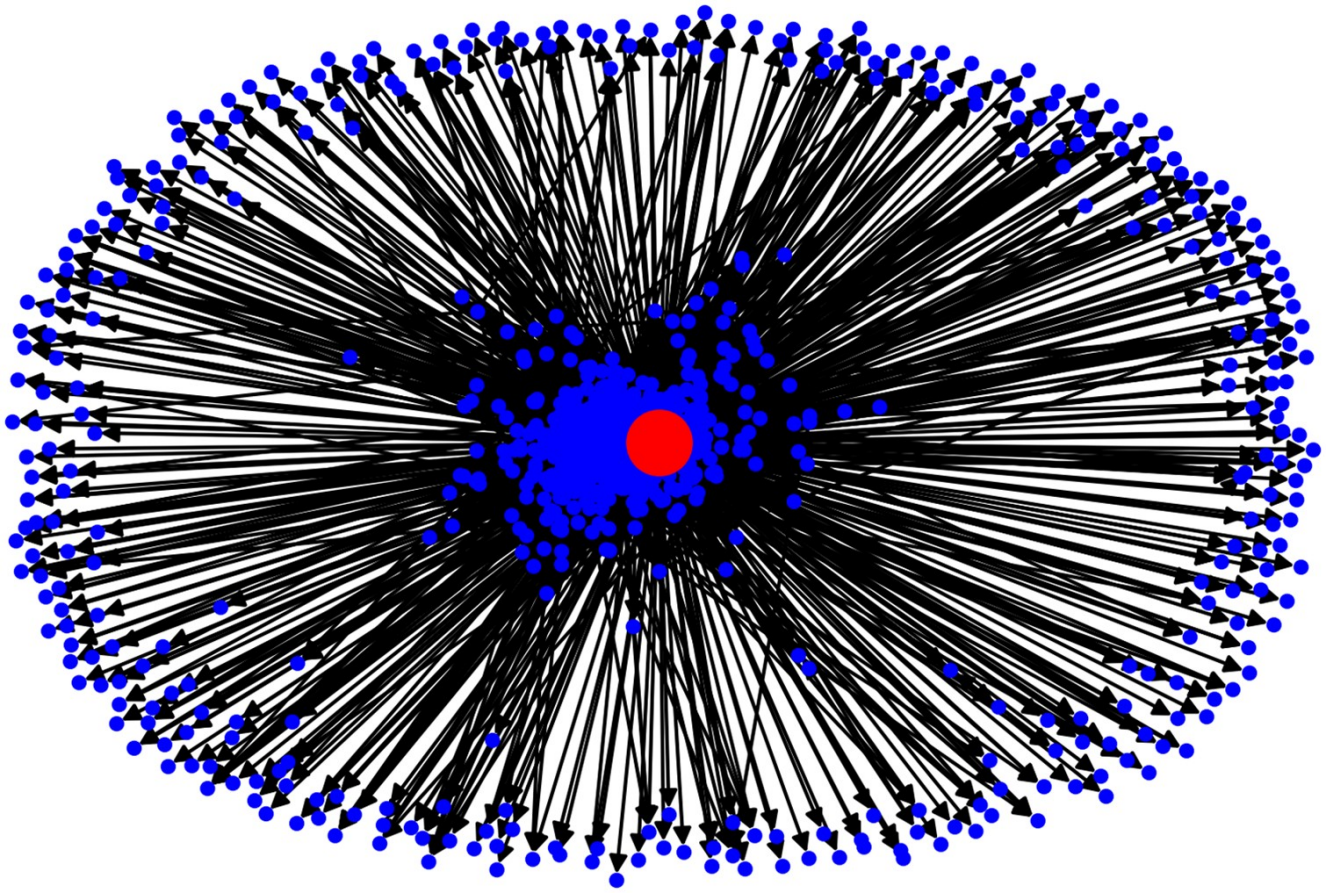
Citation network for topmost applicant ‘Siemens AG’ (best viewed in color).

In our case, the citation network among the 20 most prolific companies with regard to patents is presented in Fig 6. Companies are represented as nodes, and each edge represents the citation counts between companies. The size of the node is proportional to the citations received by the node. The network in the graph exhibits a core-periphery structure with some specific nodes acting as *authorities*. For instance, we can notice that “Siemens AG” has a lot of incoming links but no outgoing links, suggesting that while other companies in top-20 tend to cite patents from other companies, the leading ones do not necessarily cite others. This represents a hierarchy among prolific companies and makes the citations asymmetric. Also, there are companies that tend to cite other top companies a lot like for instance, “BASF AG”. This could be attributed to the fact that patent citations are often invoked due to legal issues and completeness ensured by patent examiners. On the other hand, there are companies like “Siemens AG” which are cited a lot while themselves citing very few top companies. This either reflects that such companies work in a niche area and have a *umbrella effect* on other companies or that there are certain patents held by such corporations that are essential to innovation and production of knowledge in certain areas and hence the large number of citations. Both kind of companies are helpful in disseminating knowledge throughout the network by virtue of citing other companies’ patents. In fact to realize this fact we provide in Fig 7 the periphery structure for the topmost applicant ‘SIEMENS AG’ and we can notice that there is a significant amount of citations to the central node. Due to lack of clarity in visualizing all the nodes in the graph, we could not present the plot for the complete set of nodes and edges. However, it is clear from Figs 6 and 7, that there is a peripheral structure in the network.

For the whole network we computed the list of hubs and authorities which is presented in Table 9. It is interesting to note some applicants like “Siemens AG” appear in both the top-10 hubs and authorities list and the same effect is observed in the graph of top applicants. Another interesting observation is that the graph shows a “small-world” effect where any top company can reach another with only a few steps [96]. To statistically determine this effect we followed the procedure as listed below:

- We computed the average shortest path length *L* and the clustering coefficient *C* of the network.
- We then generated an ensemble of null-model networks, including a Erdös-Rényi random graph and a Maslov-Sneppen random graph.
- Next we calculated the mean of the average shortest path length *L*_*r*_ over the ensemble of null-model networks and analogously computed *C*_*r*_.
- Finally we computed the normalised shortest path $\lambda =\frac{L}{L_{r}}$ and $\gamma =\frac{C}{C_{r}}$.

**Table 9:** Top-10 applicant hubs and authorities.

Hubs	Authorities
SIEMENS AG	SIEMENS AG
MATSUSHITA ELECTRIC IND CO LTD	IBM
GEN ELECTRIC	BAYER AG
BASF AG	PHILIPS NV
BAYER AG	MATSUSHITA ELECTRIC IND CO LTD
HOECHST AG	HOECHST AG
HITACHI LTD	BASF AG
CANON KK	HITACHI LTD
BOSCH GMBH ROBERT	CIBA GEIGY AG
SONY CORP	TOKYO SHIBAURA ELECTRIC CO
…	…

The values we achieved for λ = 0.897(≈1) and *γ* = 2.346(>1) verify the small world effect in our network and thus substantiates our hypothesis.

## Conclusions

In this study, we review patent citations within the scope of citation networks. Following the trend of existing studies, we have presented detailed descriptive analyses by indicating the top patents, the most cited patents, and the properties of the citation networks. However, in particular, we also use ERGMs to understand detailed statistical analyses of the citation formation mechanisms in the network. We demonstrate that various patent characteristics have an effect on citations. Both technical features and social processes, like homophily, preferential attachment, and transitivity can lead to the formation of citations. Particularly, our statistical analysis confirms that patents are much more likely to cite each other if patent applicants are from the same country, if patents are classified by the same category, or if patents are written in the same language. We have found that recent patents are more likely to be cited. Finally, we observed that being interdisciplinary had no the impact on patent citations. We believe that by employing ERGMs on patent citation network we are facilitating a new research avenue for future exploration by researchers to investigate ways in which existing innovation affects future innovation.

Moreover, we provide analyses of the citations among the top companies in the citation network. We observed that companies (or organizations), depending on their network positions, play different roles in the citation network. It corroborated our intuitive hypothesis that the ones receiving more citations tend to be more influential. Through our analyses, we have demonstrated that there is a significant disparity in the number of patents granted and the number of citations received by companies in any particular category. Citations between companies are shown to be asymmetrical. These are signals of hierarchy among the companies within the citation network. Most companies tend to constitute a small world where each company can be reached from another with a few steps in the citation network. Thus, in the citation network, sectors can be effectively characterized by a somewhat polycentric structure. In this structure, there appears to be a high level of cohesion within lower-level categories and a moderate level of cohesion across higher-level categories depending on the level of the IPC hierarchy.

We would like to specify that our study is by no means complete and is limited by several constraints, like assuming that sector-wise citation networks are independent, so not accounting for external influences of factors like legal issues, organizational structure of companies *etc.* that drive applicants to cite certain patents. Incorporating such factors in our study would have required assimilating a much larger dataset with information from various heterogeneous sources, which was currently out-of-scope for us. In our study we also did not consider non-EP citations, which was again dictated by our dataset limitations, where we did not have information for patents either outside of our considered time-span or generated from other patent offices. It is still a big challenge in the literature to work with patent datasets generating from multiple patent offices mostly due to mismatch in classification systems adopted by each one of them (*e.g.,* IPC vs CPC vs USPC).

Having said that, our current study is an exploratory one, and it paves the way for a set of interesting questions that are worthy of further investigation. Some of them could be directed towards investigation of the proliferation of knowledge from patents to scholarly papers and vice-versa. In particular, we would like to study the knowledge flows occurring between countries across the world and across several sectors while focusing on how the patent production in one country or geographical location affects the other both in terms of patents and scientific publications. Another prospect could be to study the evolution of citations by incorporating temporal aspects in the citation network.

To conclude, this paper provides a descriptive analysis of the patent citation network in order to describe the structural properties of the network and its implications on the patents and their citations. It also delivers a deeper analysis into the “prestige” network of top applicants, stressing on the interaction among themselves and their implications in knowledge flow. And, finally, it performs a study of the effects of various self-defined covariates on the patent citation forming mechanisms using ERGMs. The findings of this research substantiates previous works on similar lines especially in terms of homophily and sociological aspects. However, this is the first study focusing on patent citation forming mechanisms using ERGMs dealing also with the effects of factors like the influence of patent recency, overlapping and multiple categorization, the effect of cross-country patent influence, and the interaction of prolific applicants.

## References

[1] ÅKerlind Gerlese S. Academic growth and development—How do university academics experience it? *Higher Education*, 50(1):1–32, 7 2005 10.1007/s10734-004-6345-1

[2] Ladas Stephen Pericles. *Patents*, *trademarks*, *and related rights: national and international protection*, volume 1 Harvard University Press, 1975.

[3] van Raan Anthony FJ. Patent citations analysis and its value in research evaluation: A review and a new approach to map technology-relevant research. *Journal of Data and Information Science*, 2(1):13–50, 2017 10.1515/jdis-2017-0002

[4] Meyer Martin. What is special about patent citations? differences between scientific and patent citations. *Scientometrics*, 49(1):93–123, 2000 10.1023/A:1005613325648

[5] Kessler Maxwell Mirton. Bibliographic coupling between scientific papers. *American Documentation*, 14(1):10–25, 1963 10.1002/asi.5090140103

[6] Henry Small. Co-citation in the scientific literature: A new measure of the relationship between two documents. *Journal of the American Society for Information Science*, 24(4):265–269, 1973 10.1002/asi.4630240406

[7] Hirsch J. E. An index to quantify an individual’s scientific research output. *Proceedings of the National Academy of Sciences*, 102(46):16569–16572, 2005 10.1073/pnas.0507655102 16275915 PMC1283832

[8] Ivan Haščič, Nick Johnstone, Fleur Watson, and Christopher Kaminker. Climate Policy and Technological Innovation and Transfer: An Overview of Trends and Recent Empirical Results. OECD Environment Working Papers 30, OECD Publishing, 2010.

[9] Acs Zoltan J, Anselin Luc, and Varga Attila. Patents and innovation counts as measures of regional production of new knowledge. *Research Policy*, 31(7):1069–1085, 2002 10.1016/S0048-7333(01)00184-6

[10] Trajtenberg Manuel. A penny for your quotes: Patent citations and the value of innovations. *The RAND Journal of Economics*, 21(1):172–187, 1990 10.2307/2555502

[11] Fritz Foley C. and Kerr William R. Ethnic innovation and u.s. multinational firm activity. *Management Science*, 59(7):1529–1544, 2013 10.1287/mnsc.1120.1684

[12] Kerr William R. Ethnic scientific communities and international technology diffusion. *The Review of Economics and Statistics*, 90(3):518–537, 2008 10.1162/rest.90.3.518

[13] Agrawal Ajay, Kapur Devesh, and McHale John. How do spatial and social proximity influence knowledge flows? evidence from patent data. *Journal of Urban Economics*, 64(2):258–269, 2008 10.1016/j.jue.2008.01.003

[14] Singh Jasjit and Marx Matt. Geographic constraints on knowledge spillovers: Political borders vs. spatial proximity. *Management Science*, 59(9):2056–2078, 2013 10.1287/mnsc.1120.1700

[15] Thompson Peter. Patent citations and the geography of knowledge spillovers: Evidence from inventor- and examiner-added citations. *The Review of Economics and Statistics*, 88(2):383–388, 2006 10.1162/rest.88.2.383

[16] Lusher Dean, Koskinen Johan, and Robins Garry. *Exponential random graph models for social networks: Theory*, *methods*, *and applications*. Cambridge University Press, 2013.

[17] Mogee Mary Ellen. Using Patent Data for Technology Analysis and Planning. *Research-Technology Management*, 34(4):43–49, 1991 10.1080/08956308.1991.11670755

[18] Joung Junegak and Kim Kwangsoo. Monitoring emerging technologies for technology planning using technical keyword based analysis from patent data. *Technological Forecasting and Social Change*, 114:281—292, 2017 10.1016/j.techfore.2016.08.020

[19] Eleonora Pantano, Constantinos-Vasilios Priporas, Stefano Sorace, and Iazzolino Gianpaolo. Does innovation-orientation lead to retail industry growth? empirical evidence from patent analysis. *Journal of Retailing and Consumer Services*, 34:88—94, 2017 10.1016/j.jretconser.2016.10.001

[20] Radicchi Filippo, Fortunato Santo, and Castellano Claudio. Universality of citation distributions: Toward an objective measure of scientific impact. *Proceedings of the National Academy of Sciences*, 105(45):17268–17272, 2008 10.1073/pnas.0806977105 18978030 PMC2582263

[21] Leo Egghe. Theory and practise of the g-index. *Scientometrics*, 69:131–152, 2006 10.1007/s11192-006-0144-7

[22] Liebowitz S. J. and Palmer J. P. Assessing the relative impacts of economics journals. *Journal of Economic Literature*, 22(1):77–88, 1984.

[23] Molinari Jean-Francois and Molinari Alain. A new methodology for ranking scientific institutions. *Scientometrics*, 75(1):163–174, 2008 10.1007/s11192-007-1853-2

[24] Cimini Giulio, Gabrielli Andrea, and Labini Francesco Sylos. The scientific competitiveness of nations. *PLOS ONE*, 9(12):e113470, 2014 10.1371/journal.pone.0113470 25493626 PMC4262272

[25] Bakkalbasi Nisa, Bauer Kathleen, Glover Janis, and Wang Lei. Three options for citation tracking: Google scholar, scopus and web of science. *Biomedical Digital Libraries*, 3(1):7, 6 2006 10.1186/1742-5581-3-7 16805916 PMC1533854

[26] An Yuan, Janssen Jeannette C. M., and Milios Evangelos E. Characterizing and mining the citation graph of the computer science literature. *Knowl. Inf. Syst.*, 6(6):664–678, 2004 10.1007/s10115-003-0128-3

[27] Karki M.M.S. Patent citation analysis: A policy analysis tool. *World Patent Information*, 19(4):269—272, 1997 10.1016/S0172-2190(97)00033-1

[28] Criscuolo Paola, Narula Rajneesh, and Verspagen Bart. Role of home and host country innovation systems in r&d internationalisation: a patent citation analysis. *Economics of Innovation and New Technology*, 14(5):417–433, 2005 10.1080/1043859042000315285

[29] Albert M.B., Avery D., Narin F., and McAllister P. Direct validation of citation counts as indicators of industrially important patents. *Research Policy*, 20(3):251—259, 1991 10.1016/0048-7333(91)90055-U

[30] Maurseth Per Botolf and Verspagen Bart. Knowledge spillovers in europe: a patent citations analysis. *Scandinavian Journal of Economics*, 104(4):531–545, 2002 10.1111/1467-9442.00300

[31] Almeida Paul. Knowledge sourcing by foreign multinationals: Patent citation analysis in the us semiconductor industry. *Strategic management journal*, 17(S2):155–165, 1996 10.1002/smj.4250171113

[32] Hall Bronwyn H, Jaffe Adam, and Trajtenberg Manuel. Market value and patent citations. *RAND Journal of economics*, pages 16–38, 2005.

[33] Carpenter P Mark., Narin Francis, and Woolf Patricia. Citation rates to technologically important patents. *World Patent Information*, 3(4):160—163, 1981 10.1016/0172-2190(81)90098-3

[34] Fontana Roberto, Nuvolari Alessandro, Shimizu Hiroshi, and Vezzulli Andrea. Reassessing patent propensity: Evidence from a dataset of r&d awards, 1977-2004. *Research Policy*, 42(10):1780—1792, 2013 Economics, innovation and history: Perspectives in honour of Nick von Tunzelmann. 10.1016/j.respol.2012.05.014

[35] Zhang Yi, Qian Yue, Huang Ying, Guo Ying, Zhang Guangquan, and Lu Jie. An entropy-based indicator system for measuring the potential of patents in technological innovation: rejecting moderation. *Scientometrics*, 111(3):1925–1946, 6 2017 10.1007/s11192-017-2337-7

[36] Lupu Mihai, Mayer Katja, Kando Noriko, and Trippe J Anthony. *Current Challenges in Patent Information Retrieval*. Springer Publishing Company, Incorporated, 2nd edition, 2017.

[37] Lai Kuei-Kuei and Wu Shiao-Jun. Using the patent co-citation approach to establish a new patent classification system. *Information Processing & Management*, 41(2):313—330, 2005 10.1016/j.ipm.2003.11.004

[38] Park Youngjin and Yoon Janghyeok. Application technology opportunity discovery from technology portfolios: Use of patent classification and collaborative filtering. *Technological Forecasting and Social Change*, 118:170—183, 2017 10.1016/j.techfore.2017.02.018

[39] Basberg BjÃžrn L. Patents and the measurement of technological change: A survey of the literature. *Research Policy*, 16(2):131–141, 1987 10.1016/0048-7333(87)90027-8

[40] Petruzzelli Antonio Messeni. The impact of technological relatedness, prior ties, and geographical distance on university-industry collaborations: A joint-patent analysis. *Technovation*, 31(7):309–319, 2011 10.1016/j.technovation.2011.01.008

[41] Adam B Jaffe, Manuel Trajtenberg, and Michael S Fogarty. The meaning of patent citations: Report on the NBER/Case-Western Reserve survey of patentees. Technical report, National bureau of economic research, 2000.

[42] Michel Jacques and Bettels Bernd. Patent citation analysis. a closer look at the basic input data from patent search reports. *Scientometrics*, 51(1):185–201, 2001 10.1023/A:1010577030871

[43] Fortunato Santo, Bergstrom Carl T, Katy Börner, Evans James A, Helbing Dirk, Staša Milojević, et al Science of science. *Science*, 359(6379):eaao0185, 2018 10.1126/science.aao0185 29496846 PMC5949209

[44] MacRoberts Michael H. and MacRoberts Barbara R. Problems of citation analysis: A critical review. *JASIS*, 40(5):342–349, 1989 10.1002/(SICI)1097-4571(198909)40:5<342::AID-ASI7>3.0.CO;2-U

[45] Garfield E. Is citation analysis a legitimate evaluation tool? *Scientometrics*, 1(4):359–375, May 1979 10.1007/BF02019306

[46] Verena Bauer, Dietmar Harhoff, and Göran Kauermann. A smooth dynamic network model for patent collaboration data, *arXiv preprint arXiv:1909.00736*, 2019.

[47] Agrawal Ajay, Cockburn Iain, and McHale John. Gone but not forgotten: knowledge flows, labor mobility, and enduring social relationships. *Journal of Economic Geography*, 6(5):571–591, 9 2006 10.1093/jeg/lbl016

[48] Breschi Stefano and Lissoni Francesco. Mobility of skilled workers and co-invention networks: an anatomy of localized knowledge flows. *Journal of Economic Geography*, 9(4):439–468, 3 2009 10.1093/jeg/lbp008

[49] Mayank Singh, Arindam Pal, Lipika Dey, and Animesh Mukherjee. Innovation and revenue: deep diving into the temporal rank-shifts of fortune 500 companies. In *Proceedings of the 7th ACM IKDD CoDS and 25th COMAD*, pages 268–274. 2020.

[50] Kuhn J, Younge K and Marco A. Patent citations reexamined. *The RAND Journal of Economics*, 51(1):109, 2020 10.1111/1756-2171.12307

[51] An Weihua and Ding Ying. The Landscape of Causal Inference: Perspective from Citation Network Analysis. *The American Statistician*, 72(3):265–277, 2018 10.1080/00031305.2017.1360794

[52] Lusher Dean, Koskinen Johan, and Robins Garry. *Exponential random graph models for social networks: Theory*, *methods*, *and applications*. Cambridge University Press, 2013.

[53] Jackson Matthew O. *Social and economic networks*. Princeton university press, 2010.

[54] Friedkin Noah E. *A structural theory of social influence*, volume 13 Cambridge University Press, 2006.

[55] Ward Michael D, Stovel Katherine, and Sacks Audrey. Network analysis and political science. *Annual Review of Political Science*, 14:245–264, 2011 10.1146/annurev.polisci.12.040907.115949

[56] Hollway James and Koskinen Johan. Multilevel embeddedness: The case of the global fisheries governance complex. *Social Networks*, 44:281–294, 2016 10.1016/j.socnet.2015.03.001

[57] Christakis Nicholas A and Fowler James H. The spread of obesity in a large social network over 32 years. *New England journal of medicine*, 357(4):370–379, 2007 10.1056/NEJMsa066082 17652652

[58] Kretzschmar Mirjam and Morris Martina. Measures of concurrency in networks and the spread of infectious disease. *Mathematical biosciences*, 133(2):165–195, 1996 10.1016/0025-5564(95)00093-3 8718707

[59] Yang Dong-Hui and Yu Guang. Static analysis and exponential random graph modelling for micro-blog network. *Journal of Information Science*, 40(1):3–14, 2014 10.1177/0165551508088968

[60] Caimo Alberto, Pallotti Francesca, and Lomi Alessandro. Bayesian exponential random graph modelling of interhospital patient referral networks. *Statistics in medicine*, 36(18):2902–2920, 2017 10.1002/sim.7301 28421624

[61] Kim Ji Youn, Howard Michael, Pahnke Emily Cox, and Boeker Warren. Understanding network formation in strategy research: Exponential random graph models. *Strategic management journal*, 37(1):22–44, 2016 10.1002/smj.2454

[62] Newman Mark EJ, Watts Duncan J, and Strogatz Steven H. Random graph models of social networks. *Proceedings of the National Academy of Sciences*, 99(suppl 1):2566–2572, 2002 10.1073/pnas.012582999 11875211 PMC128577

[63] Newman Mark EJ and Park Juyong. Why social networks are different from other types of networks. *Physical review E*, 68(3):036122, 2003 10.1103/PhysRevE.68.036122 14524847

[64] Schweitzer Frank, Fagiolo Giorgio, Sornette Didier, Vega-Redondo Fernando, Vespignani Alessandro, and White Douglas R. Economic networks: The new challenges. *Science*, 325(5939):422–425, 2009 10.1126/science.1173644 19628858

[65] Barabási Albert-László, Albert Réka, and Jeong Hawoong. Scale-free characteristics of random networks: the topology of the world-wide web. *Physica A: statistical mechanics and its applications*, 281(1-4):69–77, 2000 10.1016/S0378-4371(00)00018-2

[66] Jeong Hawoong, Néda Zoltan, and Barabási Albert-László. Measuring preferential attachment in evolving networks. *EPL (Europhysics Letters)*, 61(4):567, 2003 10.1209/epl/i2003-00166-9

[67] Wasserman Stanley and Pattison Philippa. Logit models and logistic regressions for social networks: I. an introduction to markov graphs andp. *Psychometrika*, 61(3):401–425, 1996 10.1007/BF02294547

[68] Mark S Handcock, Garry Robins, Tom Snijders, Jim Moody, and Julian Besag. Assessing degeneracy in statistical models of social networks. Technical report, Citeseer, 2003.

[69] Snijders Tom AB, Pattison Philippa E, Robins Garry L, and Handcock Mark S. New specifications for exponential random graph models. *Sociological methodology*, 36(1):99–153, 2006 10.1111/j.1467-9531.2006.00176.x

[70] Lehmann Erich L and Casella George. *Theory of point estimation*. Springer Science & Business Media, 2006.

[71] Krivitsky Pavel N. Using contrastive divergence to seed monte carlo mle for exponential-family random graph models. *Computational Statistics & Data Analysis*, 107:149–161, 2017 10.1016/j.csda.2016.10.015

[72] Byshkin Maksym, Stivala Alex, Mira Antonietta, Robins Garry, and Lomi Alessandro. Fast maximum likelihood estimation via equilibrium expectation for large network data. *Scientific Reports*, 8(1):11509, 2018 10.1038/s41598-018-29725-8 30065311 PMC6068132

[73] Borisenko Alexander, Byshkin Maksym, and Lomi Alessandro. A simple algorithm for scalable monte carlo inference. *arXiv prxeprint arXiv:1901.00533*, 2019.

[74] Laurent Younes. Estimation and annealing for gibbsian fields. In *Annales de l’IHP Probabilités et statistiques*, volume 24, pages 269–294, 1988.

[75] Stivala Alex, Robins Garry, and Lomi Alessandro. Exponential random graph model parameter estimation for very large directed networks. *arXiv prxeprint arXiv:1904.08063*, 2019. 10.1371/journal.pone.0227804 PMC6980401 31978150

[76] Robins Garry, Pattison Pip, and Wang Peng. Closure, connectivity and degree distributions: Exponential random graph (p*) models for directed social networks. *Social Networks*, 31(2):105–117, 2009 10.1016/j.socnet.2008.10.006

[77] Thomas Kesselring. Jean Piaget, volume 512. CH Beck, 1999.

[78] Goodreau Steven M, Handcock Mark S, Hunter David R, Butts Carter T, and Morris Martina. A statnet Tutorial. *Journal of statistical software*, 24(9):1, 2008 10.18637/jss.v024.i09 18612375 PMC2443947

[79] Newman Mark EJ. The structure of scientific collaboration networks. *Proceedings of the National Academy of Sciences*, 98(2):404–409, 2001 10.1073/pnas.021544898 11149952 PMC14598

[80] Hou Haiyan, Kretschmer Hildrun, and Liu Zeyuan. The structure of scientific collaboration networks in Scientometrics. *Scientometrics*, 75(2):189–202, 2007 10.1007/s11192-007-1771-3

[81] Shi Feng, Foster Jacob G, and Evans James A. Weaving the fabric of science: Dynamic network models of science’s unfolding structure. *Social Networks*, 43:73–85, 2015 10.1016/j.socnet.2015.02.006

[82] Yan Erjia, Ding Ying, Cronin Blaise, and Leydesdorff Loet. A bird’s-eye view of scientific trading: Dependency relations among fields of science. *Journal of Infometrics*, 7(2):249–264, 2013 10.1016/j.joi.2012.11.008

[83] Shan Jiang. Statistical modeling of multi-dimensional knowledge diffusion networks: An ergm-based framework, 2015.

[84] Priem Jason and Hemminger Bradely H. Scientometrics 2.0: New metrics of scholarly impact on the social Web. *First monday*, 15(7), 2010.

[85] Cronin Blaise and Sugimoto Cassidy R. *Beyond bibliometrics: Harnessing multidimensional indicators of scholarly impact*. MIT Press, 2014.

[86] Ying Ding, Ronald Rousseau, and Dietmar Wolfram. Measuring scholarly impact. Springer, 2016.

[87] McPherson Miller, Smith-Lovin Lynn, and Cook James M. Birds of a feather: Homophily in social networks. *Annual review of sociology*, 27(1):415–444, 2001 10.1146/annurev.soc.27.1.415

[88] Siler Kyle, Lee Kirby, and Bero Lisa. Measuring the effectiveness of scientific gatekeeping. *Proceedings of the National Academy of Sciences*, 112(2):360–365, 2015 10.1073/pnas.1418218112 25535380 PMC4299220

[89] M. Singh, A. Jaiswal, P. Shree, A. Pal, A. Mukherjee, and P. Goyal. Understanding the impact of early citers on long-term scientific impact. In 2017 ACM/IEEE Joint Conference on Digital Libraries (JCDL), pages 1–10, 2017.

[90] Hunter David R. Curved exponential family models for social networks. *Social Networks*, 29(2):216–230, 2007 10.1016/j.socnet.2006.08.005 18311321 PMC2031865

[91] Papachristos Andrew V, Hureau David M, and Braga Anthony A. The corner and the crew: The influence of geography and social networks on gang violence. *American sociological review*, 78(3):417–447, 2013 10.1177/0003122413486800

[92] Barrat Alain and Weigt Martin. On the properties of small-world network models. *The European Physical Journal B-Condensed Matter and Complex Systems*, 13(3):547–560, 2000 10.1007/s100510050067

[93] Latora Vito and Marchiori Massimo. Is the boston subway a small-world network? *Physica A: Statistical Mechanics and its Applications*, 314(1-4):109–113, 2002 10.1016/S0378-4371(02)01089-0

[94] Bialonski Stephan, Horstmann Marie-Therese, and Lehnertz Klaus. From brain to earth and climate systems: Small-world interaction networks or not? *Chaos: An Interdisciplinary Journal of Nonlinear Science*, 20(1):013134, 2010 10.1063/1.3360561 20370289

[95] Ansmann Gerrit and Lehnertz Klaus. Constrained randomization of weighted networks. *Physical Review E*, 84(2):026103, 2011 10.1103/PhysRevE.84.026103 21929060

[96] Watts Duncan J and Strogatz Steven H. Collective dynamics of ‘small-world’ networks. *nature*, 393(6684):440, 1998 10.1038/30918 9623998

